# Dietary (−)-Epigallocatechin Gallate (EGCG): State-of-the-Art Advances in Bioactivities, Bioavailability Enhancement Strategies, and Applications in Nutrition and Health

**DOI:** 10.3390/nu18020317

**Published:** 2026-01-19

**Authors:** Li Niu, Yisi Luo, Weiwen Xie, Chao Wang, Zhonghua Liu

**Affiliations:** 1 Key Laboratory of Tea Science of Ministry of Education, Hunan Agricultural University, Changsha 410128, China; niuli@hunau.edu.cn; 2 National Research Center of Engineering and Technology for Utilization of Botanical Functional Ingredients, Changsha 410128, China; 3 Yuelushan Laboratory, Changsha 410128, China; 4 Xiang Tea College, Hunan Vocational College of Commerce, Changsha 410205, China; 5 Beijing Yiyeguizhen Biotechnology Co., Ltd., Beijing 100071, China

**Keywords:** (−)-epigallocatechin gallate, bioactivity, bioavailability, applications, reflections

## Abstract

(−)-Epigallocatechin gallate (EGCG), a major polyphenolic compound in tea leaves, exhibits potent antioxidant, anti-inflammatory, and anticancer properties. Despite its therapeutic potential, poor bioavailability limits clinical efficacy. This review comprehensively examines the sources, and multifunctional bioactivities of EGCG, including its antioxidant, anti-inflammatory, anticancer, cardiovascular protective, metabolic regulatory, neuroprotective, gut microbiota-modulating, and antimicrobial properties. Traditional and emerging applications of EGCG are summarized from different perspectives. Strategies to enhance bioavailability such as nanotechnology, chemical modification, and combination drug regimens are highlighted. Based on existing human clinical trials, recommendations for effective and safe dosage ranges of EGCG intake are provided. By analyzing the current situation, providing reasonable opinions and making outlooks, the clinical value of EGCG will be fully released, which will ultimately promote human health.

## 1. Introduction

Tea beverages, brewed from tea leaves, is not only steeped in rich cultural heritage but also highly valued for numerous health benefits. Extensive research in medicine, nutrition, and food science has established that (−)-epigallocatechin gallate (EGCG) as the main component plays an important role with unique chemical structure and potent biological activities [1,2].

**Figure 1:**
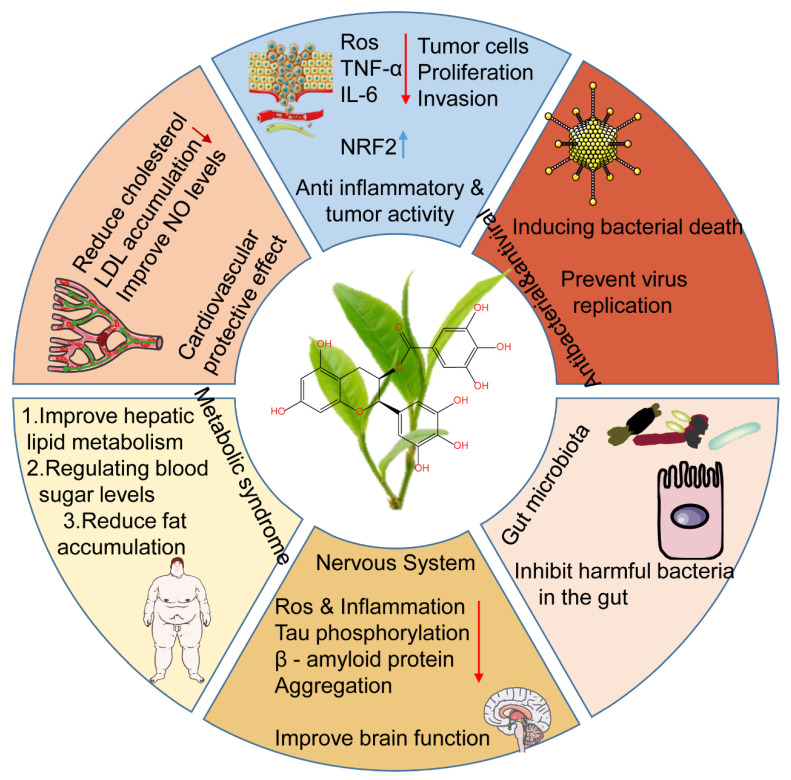
Biological activity and potential targets of EGCG. Blue arrows indicate an increase and red arrows indicate a decrease.

The molecular formula of EGCG is C_22_H_18_O_11_, formed by the connection of gallate and epigallocatechin through an ester bond. The catechin portion features a polyhydroxylated benzopyran ring, while the gallate portion consists of a benzene ring with a carboxyl group and multiple phenolic hydroxyl groups. On the one hand, the two bonding strengthens the structure of EGCG compared to epicatechin [3]. On the other hand, the multiple chiral carbon atoms in EGCG impart a specific stereochemical configuration, enabling it to effectively interact with biological targets such as enzyme active sites or cell membrane receptors to modulate cellular physiological functions [4]. These characteristics collectively confer EGCG with diverse biological activities, including antioxidant, anti-inflammatory, antimicrobial, anticancer, cardiovascular protection, metabolic regulation, neuroprotection, and gut microbiota improvement (Figure 1). Consequently, it demonstrates potential in preventing and treating related diseases ranging from aging to cancer [5,6,7,8,9].

However, the clinical potential of EGCG is severely limited by physicochemical property deficiencies such as unstable environmental factors, low bioavailability and rapid in vivo metabolism [10,11,12]. Recent advancements have shown promise in overcoming the mentioned challenges, and strategies such as chemical modification, nanotechnology, and synergistic combinations with other natural bioactives have proven effective in enhancing bioavailability and expanding clinical applications of EGCG [13,14,15,16].

Based on this, the article firstly provides a comprehensive overview of the mechanisms underlying the biological activities of EGCG and systematically summarizes the applications of EGCG in traditional medicine, food, and several emerging fields. Secondly, given the quality pain points of EGCG, innovative approaches to enhance the bioavailability of EGCG are highlighted. Finally, the challenges faced in practical application are further discussed and constructive recommendations are made. The aim is to fully explore the value of EGCG and promote its high-quality, sustainable and innovative development.

## 2. Biological Activity and Molecular Mechanism of EGCG

### 2.1. The Antioxidant and Anti-Inflammatory Activities

#### 2.1.1. Antioxidant Mechanisms

***Scavenging free radicals:*** EGCG exerts antioxidant activity to alleviate intracellular oxidative stress through multiple mechanisms. First, EGCG is rich in phenolic hydroxyl groups that neutralize reactive oxygen species (ROS) such as superoxide anion radical, hydroxyl radical, and hydrogen peroxide by transferring hydrogen atoms and single electrons [17]. This process generates stable phenoxyl radicals, thereby blocking the free radical-induced chain reaction. In addition, the phenolic hydroxyl group of EGCG chelates metal ions (e.g., Ca^2+^, Fe^2+^/Fe^3+^, and Cu^2+^) and prevents them from participating in redox reactions to reduce oxidative stress [18].

***Ameliorating Mitochondria:*** Mitochondrial damage and dysregulation are the major sources of intracellular ROS. EGCG can exert antioxidant effects by regulating mitochondrial functions. For example, it stabilizes mitochondrial membrane potential and reduces reactive oxygen species (mtROS) generation inhibiting electron leakage from respiratory chain complexes Ⅰ and Ⅲ and targeting the DRP1 apoptotic pathway [19,20,21]. EGCG also interacts directly with the OMA1 protein to inhibit mitochondrial cleavage and maintain OPA1 expression, thereby protecting structure of mitochondria [22]. Meanwhile, EGCG upregulates miR-30a expression and downregulates p53 mRNA levels to regulate mitochondrial metabolism [23].

***Enhancing the internal antioxidant defense system:*** Moreover, EGCG enhances cellular resilience against damage from external factors by reinforcing the endogenous antioxidant defense system [24]. For instance, EGCG has been shown to activate the Keap1/P62/Nrf2 signaling pathway, which leads to the upregulation of endogenous antioxidant enzymes, such as superoxide dismutase, catalase, and glutathione peroxidase, indirectly diminishing the levels of intracellular oxygen free radicals [25]. Not only that, EGCG can also achieve purpose by regulating the expression of related signaling pathway genes such as Keap1/Nrf2/ARE, PI3K/AKT, NF-κB, MAPK and protein kinases [26,27,28].

#### 2.1.2. Anti-Inflammatory Mechanisms

***Inhibiting inflammatory signaling pathways:*** EGCG exhibits anti-inflammatory effects by modulating inflammatory signaling pathways. For example, it protects microglia from hypoxia-induced inflammation and oxidative stress by inhibiting the NF-κB pathway and activating the Nrf-2/HO-1 pathway [29]. EGCG also attenuates lipopolysaccharide (LPS)-induced hepatic inflammation and mitigates inflammatory responses in cardiomyocytes exposed to cigarette smoke by inhibiting the MAPK/NF-κB signaling pathway [30,31]. Meanwhile, activation of the key inflammatory pathway cGAS/STING by EGCG spared nucleated cells from hydrogen peroxide-induced apoptosis and inflammation as well as also inhibits mtDNA release [32,33].

***Inhibiting inflammation-related mediators:*** EGCG exerts anti-inflammatory activity by effectively inhibiting the NF-κB signaling pathway, down-regulating the expression of pro-inflammatory cytokines (e.g., TNF-α, IL-1β, and IL-6) [34], and blocking the receptor activator of NF-κB ligand-induced production of TSLP, IL-1β, IL-6, and IL-8 to reduce release of inflammation factors [35].

***Modulating immune cell function and chemokine expression:*** Chemokines are crucial for the recruitment and migration of inflammatory cells. EGCG specifically inhibits the expression of chemokines, such as monocyte chemoattractant protein-1 and macrophage inflammatory protein-2 [36]. Interestingly, the trend toward suppressed M1 polarization and enhanced M2 polarization was observed in liver, colon, spleen, and brain tissues after EGCG treatment, highlighting the anti-inflammatory pathway mediated by EGCG through immune cell function regulation [37].

***Activating autophagy:*** Autophagy and inflammation are both crucial physiological processes within cells, and they share a complex interrelationship. Research has demonstrated that EGCG can activate the autophagy pathway to degrade high mobility group box 1 protein, thereby inhibiting the activation of the inflammasome in macrophages [38]. Additionally, EGCG also restores autophagy levels, suppresses the activation of the NLRP3 inflammasome by inhibiting the mammalian target of rapamycin signaling pathway [39].

#### 2.1.3. Antioxidant and Anti-Inflammatory Synergy

Since the accumulation of excess intracellular ROS often serves as an inflammatory trigger, EGCG is usually accompanied by anti-inflammatory effects in the antioxidant process. For instance, in a model of acute pancreatitis-induced lung injury, EGCG has been shown to reduce the production of mtROS and oxidized mitochondrial in alveolar macrophages, effectively suppressing the activation of theNLRP3 inflammasome [40]. In the context of DSS-induced colitis, EGCG activates the Nrf2-GPX4 pathway to enhance antioxidant defenses and concurrently upregulates ferritin to improve iron metabolism, thereby counteracting ferroptosis in colonic epithelial cells [41]. Additionally, EGCG attenuates neuroinflammation in LPS/AβO-stimulated microglia by inhibiting the ROS/TXNIP/NLRP3 axis [42]; and blocks *Staphylococcus aureus*-induced NLRP3 activation via the ROS/MAPK pathway [43].

### 2.2. The Anticancer Activity

#### 2.2.1. Inhibiting Cancer Cell Growth

With increasing research, EGCG has been shown to target cancer features through multiple mechanisms, demonstrating potent anticancer activity and potential [44]. On the one hand, EGCG can downregulate the expression of cell cycle-related genes and enhance the expression of cell cycle inhibitory factors to induce cell cycle arrest and inhibit tumor cell proliferation. Recent studies have also issued corroboration that EGCG inhibits CCL5-stimulated lung cancer cell proliferation by down-regulating Nrf2 expression [45].

#### 2.2.2. Inducing Apoptosis in Cancer Cells

As a form of programmed cell death, apoptosis is a key mechanism in cancer therapy, and EGCG has been shown to modulate the apoptotic process in various cancer types. In particular, EGCG activates the mitochondrial apoptotic pathway by downregulating SIRT1 expression to modulate the SIRT1-p53 axi [46]. In breast cancer, EGCG induces apoptosis by inhibiting miR-25 expression and elevating PARP, pre-caspase-3 and pre-caspase-9 protein levels [47]. Additionally, in multiple myeloma, EGCG promotes apoptosis by activating the endoplasmic reticulum stress pathway and disrupting the integrity of the cytoskeleton [48,49]. EGCG induces autophagic cell death in breast cancer cells by retaining YAP1 in the cytoplasm and promoting the assembly of the CHMP2B-VPS4B complex [50]. Notably, EGCG can also enhance the sensitivity of liver cancer cells to copper by activation of the MTF1/ATP7B axis to promote intracellular copper accumulation [51].

#### 2.2.3. Inhibiting Tumor Metastasis

Tumor metastasis is a leading cause of treatment failure in cancer patients, and epithelial-mesenchymal transition (EMT) is a crucial step in this process. EGCG has been demonstrated to inhibit EMT, invasion, and migration by blocking the TGFβ/Smad signaling pathway or upregulating the transcription factor EB and promoting its nuclear localization [52,53]. On the other hand, EGCG can directly bind to STAT3, reducing nuclear localization and inhibiting the transcription of PLXNC1. This action also reverses the M2 polarization induced by PLXNC1-mediated exosomes, thus suppressing the migration and invasion of gastric cancer cells [54].

#### 2.2.4. Inhibiting Angiogenesis and Vasculogenic Mimicry

Tumor cells often rely on angiogenesis and vasculogenic mimicry to obtain the nutrients necessary for their growth and spread. It is widely believed that EGCG can block this process by reducing the expression of vascular endothelial growth factor, a key factor in angiogenesis, thereby decreasing the formation of new blood vessels by endothelial cells [55]. The inhibition of angiogenic mimicry by EGCG through the Twist/VE-calmodulin/AKT pathway has also been demonstrated in prostate cancer cells [56].

#### 2.2.5. Acting as an Immunomodulator

Tumor immunotherapy represents an innovative approach that leverages the immune system to target and eliminate cancer cells. Immune checkpoint inhibitors such PD-1/PD-L1 have shown promise in clinical settings. The natural compound EGCG can be used as an immunomodulator and immune checkpoint blocker for the treatment of tumors. For example, EGCG targets the JAK-STAT signaling pathway to reduce the expression levels of downstream PD-L1/PD-L2. This reduction promotes the interaction between melanoma cells and cytotoxic T-cells, thereby enhancing the anti-tumor immune response [57]. EGCG also can down-regulate PD-1 expression in T cells by inhibiting NF-κB phosphorylation and nuclear translocation, thereby enhancing the anti-tumor killing ability of immune cells [58]. Further, EGCG modulates antitumor immunity by inhibiting M2 polarization of tumor-associated macrophages or promoting immune responses in cytotoxic lymphocytes and dendritic cells [59,60].

#### 2.2.6. Interaction with the Epidermal Growth Factor Receptor (EGFR)

Particularly considering that EGFR overexpression or mutation is closely associated with proliferation and metastasis in various cancers, extensive research has also confirmed that EGCG possesses the ability to interact with EGFR and inhibit activity, strengthening the anticancer evidence for EGCG [61,62]. Notably, EGCG not only inhibits wild-type EGFR but also suppresses activated mutant EGFR in lung cancer cells.

### 2.3. The Cardiovascular Protection Activity

In recent years, the cardiovascular protective effects of EGCG have garnered significant attention in medical research. First, the antioxidant properties of EGCG are essential for the prevention of atherosclerosis by reducing the accumulation of oxidized low-density lipoprotein (LDL) in vascular endothelial cells, which is the major pathological basis of cardiovascular disease [63]. In addition to antioxidant effects, Luo et al. [64] reported that EGCG can reduce total cholesterol and LDL-cholesterol levels in the blood while increasing high-density lipoprotein cholesterol levels to improve blood lipid profiles and prevent cardiovascular diseases. Moreover, EGCG promotes vasodilation and improves vascular reactivity by enhancing the ability of cells to produce nitric oxide, which has a positive effect on blood pressure regulation [65,66].

The significant global health burden of myocardial ischemia has spurred investigations into natural remedies as potential therapeutic options. EGCG directly acts on the heart through the 450b-5p/ACSL4 pathway to inhibit ferroptosis, thereby alleviating acute myocardial infarction [67]. Additional studies have shown that EGCG pretreatment protects cardiomyocytes by reducing iron death, apoptosis, and autophagy through upregulation of 14-3-3η protein levels [68]. During reperfusion therapy, EGCG attenuates oxidative stress and inhibits the inflammatory response to prevent further damage to cardiomyocytes [69].

### 2.4. The Metabolic Regulatory Activity

Recent studies have shown that EGCG exerts its metabolic activity through multiple mechanisms, demonstrating the potential to prevent and treat metabolic diseases. Three typical aspects of regulating blood glucose levels, improving lipid metabolism and protecting liver function are described.

Firstly, EGCG enhances blood glucose regulation by promoting insulin sensitivity and insulin signaling [70]. Secondly, EGCG also slows down the absorption of carbohydrates by inhibiting certain digestive enzymes, which is crucial for controlling postprandial blood glucose spikes [71]. Regarding lipid metabolism, EGCG promotes the degradation of fat mass and obesity-related proteins (FTO), inhibits FTO-mediated oxidative stress processes, and protects pancreatic β-cells from excessive autophagy induced by nuclear receptor subfamily 3C member 1 [72]. Simultaneously, it can reduce fat accumulation in the body by inhibiting the conversion of preadipocytes to mature fat cells [73]. It also activates thermogenesis and fat oxidation to increase energy expenditure for weight control and obesity management [74]. EGCG improves hepatic lipid metabolism and reduces hepatic fat accumulation, which has a significant protective effect on liver health. It is commonly used in the prevention and treatment of nonalcoholic fatty liver disease [75].

### 2.5. The Neurological Regulatory Activity

The role of EGCG in neuromodulation is particularly noteworthy, especially in neuroprotection and cognitive function improvement. Its neuroprotective effect is primarily attributed to antioxidant, anti-inflammatory properties, and the ability to inhibit the progression of neurodegenerative diseases, which provides new perspectives for the treatment of diseases such as Alzheimer’s disease and Parkinson’s disease, and suggests a potential natural pathway for the prevention of age-related cognitive decline.

Firstly, excessive free radicals are key contributors to cell damage and neurodegenerative diseases [76]. Through potent antioxidant activity, EGCG reduces oxidative stress and protects neurons from damage, thereby delaying neurodegeneration. Secondly, EGCG inhibits the production of inflammatory mediators, reducing neuroinflammation and thus alleviating neuronal damage and death.

In the context of Alzheimer’s diseases, the accumulation of β-amyloid protein is a central pathological feature. EGCG has been shown that not only inhibits the formation and aggregation of β-amyloid, but also regulates the aberrant phosphorylation of Tau protein, another critical factor involved in neurofibrillary tangles [77]. EGCG has also demonstrated neuroprotective effects in models of Parkinson’s disease. In short, EGCG restores iron homeostasis by inhibiting iron influx through the suppression of Malvolio expression. It also downregulates the expression of NADPH oxidases, thereby reducing the production of ROS and increasing the activity of superoxide dismutase [78].

### 2.6. The Intestinal Microecology Regulatory Activity

The intestinal flora, composed of billions of microorganisms, is closely related to human health. EGCG exerts positive effects on the intestinal tract through multiple mechanisms such as regulating the composition of the flora, host immune response and intestinal barrier function.

Expanding on this, EGCG can regulate the balance of gut flora. For example, EGCG can inhibit the growth of harmful bacteria such as *Escherichia coli* and *Salmonella*, while promoting the proliferation of probiotics like *Bifidobacterium* and *Lactobacillus* [79]. EGCG can counteract the reduction in *Clostridium* abundance caused by a high-fat diet and increase the abundances of *Deltaproteobacteria* and *Epsilonproteobacteria* [59]. On the other hand, EGCG helps to maintain the stability of the gut microbiota to reduce gut infections and inflammation caused by pathogens. Increasing the production of short-chain fatty acids by promoting the growth of probiotics directly benefits gut health and overall immune function [80]. The topic of novelty also stated that EGCG alleviates intestinal inflammation by inhibiting the production of inflammatory mediators and modulating related signaling pathways [81], and enhances the defense against pathogens by influencing mucosal immune cells to further modulate the immune response [82].

### 2.7. Antibacterial and Antiviral Properties of EGCG

EGCG has been extensively studied and proven to possess remarkable antibacterial and antiviral properties based on cell testing. This is attributed to the polyphenolic hydroxyl group in structure, which interacts with a wide range of biomolecules of bacteria and viruses, thereby inhibiting their activity or destroying their structure [83].

In antibacterial research, EGCG has demonstrated inhibitory effects on a bacterial strain, including some drug-resistant bacteria. It binds to the cell walls and membranes of bacteria to alter their structures and functions, thus inhibiting bacterial growth or directly leading to bacterial death [84]. Additionally, EGCG can interfere with the formation of bacterial biofilms, which are protective structure that help bacteria resist attacks from antibiotics and the host immune system [85]. By disrupting biofilms, EGCG enhances the effectiveness of traditional antibiotics and may help address the growing problem of drug resistance in clinical practice.

In the antiviral field, the mechanisms of action of EGCG are equally diverse. It can interfere with various stages of the viral life cycle, including viral adsorption, penetration, replication, assembly, and release. For example, EGCG can directly bind to the glycoproteins on the viral surface to hinder the interaction between the virus and the receptors on the surface of host cells, thereby preventing viral entry [86]. Research by Wang et al. [87] also indicates that EGCG can stop viral replication by inhibiting enzymes required for viral RNA or DNA replication. This action not only reduce the viral infection rate but also alleviate the cell damage caused by the virus.

## 3. Applications of EGCG

### 3.1. Traditional Medicine

**Figure 2:**
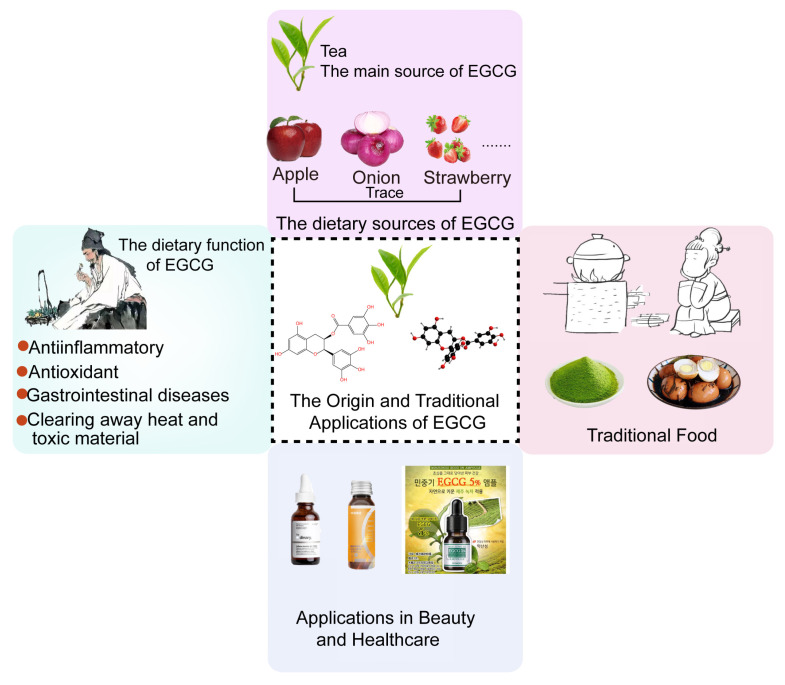
The traditional applications of EGCG. The non-English text in the image indicates existing product descriptions.

In traditional Chinese medicine (TCM), tea leaves possess a bitter and sweet flavor and enters the heart, lung, and stomach. Due to effects of invigorating the mind, detoxifying, clearing heat, and promoting digestion, they have been used as herbal remedies since ancient times (Figure 2) [88]. Recent scientific research has elucidated the specific biological activities of EGCG, providing a modern scientific basis for traditional application of tea leaves. EGCG has significant antioxidant and anti-inflammatory effects align with TCM’s concept of “clearing heat and detoxifying”. Additionally, EGCG inhibits tumor cell growth and induces apoptosis, providing support for TCM use of tea leaves in cancer prevention and adjunctive therapy. Regarding the digestive system, EGCG can alleviate gastrointestinal inflammation, which is consistent with the traditional use to aid digestion and treat indigestion. Overall, as the primary component of tea leaves, EGCG perfectly embodies the fusion of tradition and modernity.

### 3.2. Applications in Traditional Foods

In traditional diets, the cultural and health significance of tea beverages is reflected in various dietary customs and social occasions. For example, in China and Japan, drinking tea after meals is valued not only for fragrant taste but also for digestive and palate-refreshing properties. Surprisingly, EGCG has been incorporated into traditional recipes to deliver health benefits, especially in Asian countries. In Japan, matcha (a finely ground green tea powder) enjoys widespread popularity, serving not only as a core element in tea ceremonies but also as an ingredient in diverse dishes like matcha ice cream, cakes, and ramen. In China, tea leaves are similarly used in cooking, such as tea-scented chicken and tea-dipped eggs. This culinary diversity makes the intake of EGCG more diverse and interesting. With further research, EGCG’s application in both traditional and modern cooking may expand, leading to the development of more EGCG-rich foods and beverages that leverage its health benefits.

### 3.3. Cosmetics and Healthcare

Based on proven topical efficacy, EGCG is gaining increasing recognition in the field of skin anti-aging. It mitigates signs of skin aging by increasing antioxidant enzymes, inhibiting the expression of skin matrix metalloproteinases, promoting collagen synthesis, and maintaining normal dermal structure. For example, pretreatment with EGCG significantly reduces skin wrinkles, erythema, and elastic fiber damage in nude mice following ultraviolet exposure [89]. Topical application of EGCG significantly alleviates reduced skin thickness and scratching behavior in mice with atopic dermatitis, which is dependent on the ability of EGCG to significantly reduce or block the expression of inflammatory cytokines and associated kinases [90].

Meanwhile, the effects of EGCG in inhibiting type 2 diabetes [91], non-alcoholic fatty liver disease [92], and neurological disorders [93] have also been demonstrated in mouse models, providing important references for its potential as a health-promoting ingredient.

## 4. Methods to Enhance the Bioavailability of EGCG

**Figure 3:**
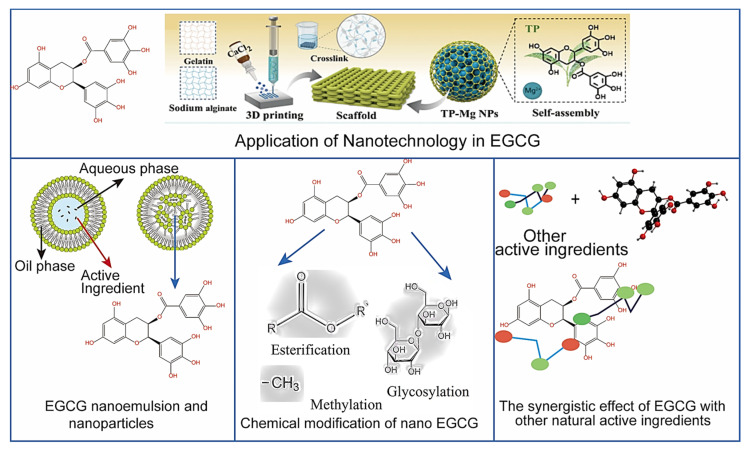
Methods to enhance the bioavailability of EGCG.

Despite the broad potential in health of EGCG, its low bioavailability in the human body limits clinical efficacy. Current research is exploring ways to enhance the bioavailability of EGCG through nanotechnology, modification techniques and combination drug programs (Figure 3).

### 4.1. Nanotechnology

**Figure 4:**
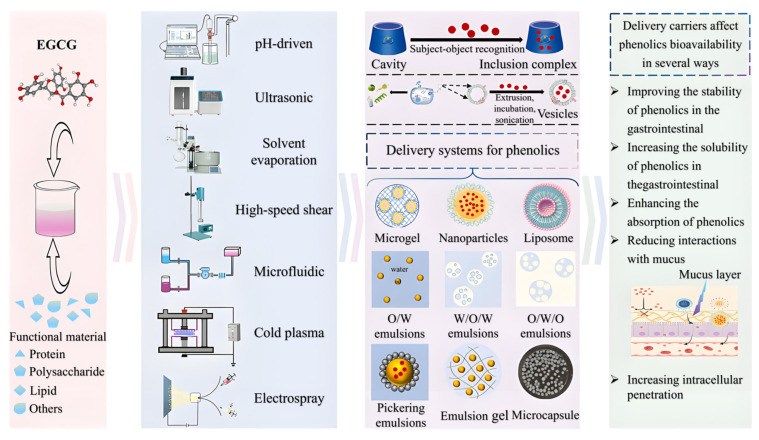
Nanotechnology strategy of EGCG.

Oral delivery based on human compliance is the preferred option, but transporting EGCG from the harsh environment of the gastrointestinal tract to systemic circulation presents many challenges. At present, the actual use of EGCG in the human body is mainly in the form of traditional capsules and tablets taken with water, whose problems of limited solubility, unstable oral utilization, and uneven distribution in body fluids remain unresolved. The transformative strategy of preparing EGCG into nanomaterials using advanced carriers has attracted attention for the ability to increase their solubility, enhance their digestive stability, bring them closer to the scale of biomolecules (<500 nm) to penetrate biological barriers, prolong the diffusion time through sustained release, and specifically target organs [94]. Han et al. [95], Minnelli et al. [96], Mehmood et al. [97], Zhang et al. [98] and Dai et al. [99] outlined this, which mainly included nanoemulsions, nanoparticles, liposomes, inclusions, nanofibers, nanomicelles, nanosheets, gels, and nanovesicles, as shown in their morphology and preparation methods in Figure 4. Physical field-driven self-assembly as well as emerging technologies are being developed integrally. There is a broad range of carrier materials, involving natural substances, synthetic substances, and metals/oxides. Many other nanosystems such as plant-derived nanoparticles and virus-like nanoparticles are also being investigated, and details about their types, properties, and delivery applications please see the recent review coverage. They can increase bioavailability by several to more than a dozen times compared to the oral bioavailability of free EGCG, thereby exhibiting more pronounced cytotoxic potential against diseases such as diabetes and cancer. It should be emphasized that supplement formulations are quite different from oral administration, as the selection of supplement carriers requires careful consideration of long-term edibility and market prices in the food category. Therefore, substances such as proteins and lipids may be preferable, as reported by Tang et al. [100] and Tang [101].

### 4.2. Chemical Modifications

In recent years, methods that enhance the stability and bioavailability of EGCG by introducing ester, methyl, and glycosyl groups into the molecular structure to alter its physical and chemical properties have garnered significant attention. Firstly, introducing hydrophobic fatty acids and methyl groups as well as hydrophilic glycosyl groups into the EGCG molecule significantly improves its biphasic solubility, thereby enhancing its ability to penetrate cell membranes and increasing uptake [102,103]. Secondly, comparing EGCG before and after modification reveals that the introduction of three functional groups significantly enhances the stability of EGCG in the gastrointestinal tract while reducing the likelihood of rapid metabolism and excretion [99,104]. Finally, taking esterified EGCG as an example, the fact that its accumulation in tumor tissues significantly increases and antitumor efficacy markedly enhances demonstrates that modification can also achieve targeted delivery of EGCG. This reduces drug distribution in non-targeted tissues, thereby minimizing potential side effects [105]. Covalent binding of EGCG to other molecules or polymers to enhance stability and bioavailability is also commonly used. For example, binding of EGCG to polyethylene glycol significantly slows its degradation [16]; binding of EGCG to hyaluronic acid enhances its water solubility and gastrointestinal absorption efficiency [97]; and binding to specific targeting molecules, such as antibodies or ligands, results in the enrichment of EGCG in specific tissues or cells [106].

### 4.3. Compatibilizer Synergy

Leveraging the compatibility of polyphenolic compounds has emerged as a promising strategy to improve the bioavailability of EGCG. Co-encapsulation assay of EGCG with quercetin shows that the two synergistically enhanced the antioxidant capacity of EGCG [107]. Combining EGCG with resveratrol increases its solubility and significantly improves its absorption in the small intestine. This combination also activates the AMPK signaling pathway and reduces the synthesis of total cholesterol and triglyceride in hepatocytes, and thus regulates the lipid metabolism [108,109]. Encouragingly, studies have also indicated that the combination of EGCG and curcumin inhibits the activity of metabolic enzymes, reduces the rate of metabolism in the liver and enhances its antitumor efficacy [110]. Moreover, the contribution of lipids cannot be overlooked. Lipids compatibility can shield EGCG from environmental fluctuations. For example, non-covalent complexes formed between EGCG and phospholipids exhibit significantly improved stability in simulated gastrointestinal conditions, with a notably reduced degradation rate and extended effective action time [111]. This is achieved by encapsulating EGCG to minimize direct exposure to the environment. Given that low water solubility of EGCG is primary factor limiting biological applications, hydrophobic microenvironment can be significantly enhanced through non-covalent interactions with lipids such as fatty acids. For example, complexes formed between EGCG and fatty acids can generate mixed micelles in the gastrointestinal tract, thereby increasing the solubility of EGCG and absorption efficiency [111].

The application of EGCG as a pharmaceutical excipient is commonly observed in cancer treatment, showing significant synergistic effects when combined with anticancer drugs. For example, the combined use of EGCG and doxorubicin had not only demonstrated encouraging results in enhancing anticancer activity, but also mitigated the side effects associated with high-dose doxorubicin monotherapy [112]. Furthermore, the combination of EGCG with the EGFR inhibitor erlotinib has been demonstrated to promote Bim-mediated apoptosis in head and neck cancer cells [113]. The synergistic effects of EGCG combined with chemotherapeutic agents such as 5-fluorouracil, celecoxib, cisplatin, and tamoxifen have also been reported by Wang et al. [44], effectively countering cancer cell resistance and enhancing drug sensitivity. This finding highlights the potential of EGCG to enhance the efficacy of existing cancer treatments, offering prospects for expanding its application across broader medical fields.

## 5. Dosage and Toxicity Analysis Based on Human Clinical Trials

Human clinical trials serve as the standard for evaluating effective and safe doses of EGCG supplements. The meta-analysis on weight loss concludes that daily intake of ≥300 mg EGCG yields statistically significant but modest effects on weight and body fat reduction (average weight loss of 1–2 kg) [114]. Higher doses (e.g., 856 mg daily) demonstrate greater efficacy in short-term studies [115]. Daily doses of 150~600 mg EGCG show a trend toward mild improvements in blood pressure, LDL-cholesterol, and insulin sensitivity, though results are not entirely consistent [116]. Trials targeting cognitive health typically employ 600~1200 mg daily, though evidence strength remains limited [117]. However, the toxicity of EGCG, particularly hepatotoxicity exhibits a clear dose-dependent pattern, which is the primary concern limiting high-dose application. The European Food Safety Authority notes in scientific opinion that daily oral doses of 800 mg or higher of EGCG represent a common starting point for observed cases of liver injury [118]. Most severe cases are associated with the consumption of high-purity EGCG or tea extract supplements, rather than drinking tea. A comprehensive analysis of existing literature indicates that the dosage range for positive results in human trials is between 150 mg and 800 mg daily, typically administered as standardized tea extract (containing approximately 50–90% EGCG) [119,120]. Based on recommendations from the European Food Safety Authority and the U.S. Pharmacopeia, along with variations in product formulation (single-ingredient supplements with high purity and high doses carry greater risks than complex tea extracts or tea leaves themselves) and physiological metabolism, a daily intake of 300–800 mg of EGCG is considered optimal, though the effective dose may be lower. Risk minimization strategies are to prioritize EGCG intake through tea consumption and avoid indiscriminate use of high-dose supplements. If supplements are necessary, they should be taken with meals. It is recommended to start with a low dose and monitor for any adverse symptoms. Individuals with a history of liver disease, pregnant women, and breastfeeding women should not use if possible.

## 6. Limitations and Recommendations in EGCG Application

The vast majority of pharmacokinetic studies on EGCG have been conducted solely in animal models. Only Veregen^®^ has received FDA approval for clinical treatment of genital warts and human papillomavirus [121,122]. In addition to the aforementioned factors, the auto-oxidation of EGCG renders it a pro-oxidant, generates quinone-type oxidation products, impairs the activity of drug-metabolizing enzymes, disrupts signaling pathways, and damages the liver [123,124]. Meanwhile, the nonlinear nature of auto-oxidation reactions implies that the window between safe and toxic doses may be narrower than anticipated. This is the primary reason for the confusion, inconsistency, difficulty in replication, and overinterpretation of trial results. Furthermore, EGCG is frequently incorporated into functional foods due to purported health benefits. However, the current lack of a standardized regulatory framework governing content, efficacy, and safety of EGCG has led to inconsistent product quality and varying efficacy claims in the market, thereby increasing regulatory challenges.

Therefore, firstly, a reasonable regulatory system needs to be established to create a favorable business atmosphere. Secondly, attention should be paid to the assessment of the safety and efficacy of EGCG in clinical application, aiming at the formulation of standardized use guidelines and dosage recommendations to ensure scientific application in clinical practice. It is essential to distinguish whether the toxicity originates from EGCG or auto-oxidative products. Auto-oxidation conditions must be actively controlled and reported in trial designs (e.g., using antioxidants, freshly preparing samples, controlling pH and time, measuring residual concentrations, etc.), with rigorous chemical controls established. Finally, there is an urgent need to expand the evidence supporting safe and effective clinical translation and to determine its general applicability through large-scale population trials.

## 7. Conclusions and Future Perspectives

As a multifunctional natural compound, EGCG has garnered significant attention for its remarkable roles in health maintenance and disease prevention and treatment. By integrating the existing scientific studies, the structural and physicochemical properties of EGCG, its origin, functional properties, application areas, methods to enhance its bioavailability, and recommendations for safe and effective dosages have been clarified. Looking forward, the extraction, purification and synthesis technologies of EGCG as well as the enhancement of bioavailability should be further optimized. Meanwhile, standardized usage guidelines and dosage recommendations should be formulated to ensure its scientific application in clinical practice. Through interdisciplinary cooperation, a comprehensive understanding of action mechanism for EGCG in complex biological systems will provide a solid scientific foundation for the development of new drugs and health products.

## Data Availability

No new data were created or analyzed in this study. Data sharing is not applicable to this article.
